# Modelling of Resinous Material Filling Expansion Joints in Reinforced Concrete Structures

**DOI:** 10.3390/ma16052011

**Published:** 2023-02-28

**Authors:** Krzysztof Schabowicz, Grzegorz Waśniewski, Krzysztof Wróblewski

**Affiliations:** Faculty of Civil Engineering, Wrocław University of Science and Technology, Wybrzeże Wyspiańskiego 27, 50-370 Wrocław, Poland

**Keywords:** reinforced concrete structure, dilatation gap, *FEM* model, hyperelasticity

## Abstract

This paper is a continuation of the research and analysis to estimate hyperelastic material constants when only uniaxial test data are available. The FEM simulation was expanded and the results obtained from three-dimensional and plane strain expansion joint models were compared and discussed. The original tests were carried out for a gap with a width of 10 mm, whereas in the case of axial stretching, the stresses and internal forces caused by the leading deformations were recorded for a smaller gap, and the axial compression was also recorded. The differences in the global response between the three- and two-dimensional models were also considered. Finally, using FEM simulations, the values of stresses and cross-sectional forces in the filling material were determined, which can be the basis for the design of expansion joints geometry. The results of these analyses could form the basis of guidelines for the design of expansion joint gaps filled with material, ensuring the waterproofing of the joint.

## 1. Introduction

Building construction represents one of the largest development uses of construction chemicals. Big buildings are divided into parts that are separated by expansion joints. These are usually made during the construction and are present in every element, from the bottom slab to the roof structure [1,2,3,4,5,6,7].

The main problem for the system of filling the expansion joint is water, because it causes damage to internal installations and its structure. One of the most frequent problems is water leakage through expansion joints. This is a real problem in buildings under construction and during their service life [8,9,10]. Therefore, the material filling the expansion joint should ensure its waterproofness. In this case, application of a polyurethane-based resin is a good choice [9,10,11,12].

Within the expansion joints, section forces occur caused by, i.a., thermal deformations, concrete creep and shrinkage, and the uneven subsidence of the structural members [12,13]. Various fillings, sealing materials, and closing in the form of premoulded inserts and sealing strips are applied to ensure the expansion joints’ waterproofness [11] against the above-mentioned non-mechanical excitations.

There are several products on the market for sealing expansion joints selected on the basis of “the engineering knowledge, experience and assurances of the sealing crews”. One of them—the resin—becomes a permanent flexible mass after curing, which, during the cyclic excitations acting on the expansion joint, should expand or shrink depending on changes in the geometry of the expansion joint. There are no standards and guidelines that would indicate the use of a particular filler under specific conditions. There are also no standards and guidelines for surface preparation, i.e., the preparation of sidewall surfaces in an expansion joint [14,15]. For this reason, the comparison of the results of the FEM numerical analysis and the experimentally determined material strength parameters seems to be the right direction for further research in order to correctly design the optimum width of the expansion joint.

The authors of [16] presented an interesting alternative to other ways of fixing adhesive bonded joints between glass panels and their load-bearing metal structures in façade constructions. Silicon sealants have been studied for their excellent adhesion to glass and exceptionally high resistance to environmental influences and aging. *FEM* nonlinear numerical simulations were used to verify filled joints. An overview of the available damage criteria for rubber-type materials was presented. The criteria application to silicone sealants was verified for three characteristic stress states: uniaxial tension, compression, and shearing.

A polyurethane polymer, which owing to specially selected additives providing a better damping, was analyzed in PhD thesis [17]. The efficiency of a novel material for a seismic vibration isolation bearing was carried out by experimental and detailed nonlinear numerical *FEM* analyses. Discrete models response analysis of existing steel structure with and without vibroisolation to several seismic and paraseismic excitations were performed. Laboratory tests (compression and tension) allowed the determination of the material constants for a five-parameter Mooney–Rivlin model for the analyzed polymer material.

In [18], the authors considered elastomeric tracks for industrial vehicles, in which materials are incompressible and very high deformable. The material parameters for several hyperelastic material models (Mooney–Rivlin) were determined in experiments. The parameters were used to define *FEM* discrete models for computations. Numerical results obtained for different models were compared with experiments, in which the samples were exposed to the same load.

This manuscript is a description of one of the stages, into which the authors have divided the study of the issue. The first step proposes a novel way to identify a physical model of a hyperelastic material, where only a limited set of experimental data is available. The results of these investigations were published in [19]. The next stage, presented in this manuscript, concerns the possibility of numerically determining stress distributions for typical excitations, to which the material filling the expansion joint is subjected. In this part, the authors want to assess whether, in regard to the identified material model and its previously determined physical constants, numerical simulations will give results in acceptable ranges. In the final stage, for numerically determined distributions of the stress tensor components, it is intended that various damage criteria will be applied, and attempts to verify them experimentally will be made.

## 2. Objectives

The aim of the development and continuation of the research is to carry out *FEM* simulations for a resin material in an expansion gap under different non-mechanical excitations: axial stretching and compression, bending, and shearing. The influence of the width of the gap on the response of the filling material in dilatated reinforced concrete beams and slabs at different levels of forcing is studied. The results are the values of stresses and cross-sectional forces in the filling material, which can be the basis for the design of expansion joints geometry.

As a material filling the expansion joint, polyurethane-based resin was considered. Its description was given in Section 3. It should be noted that during the deformation process, material can be subjected to extreme strains, even more than 100% in the case of stretching. Moreover, during laboratory tests, one observed that deformations are reversible in the whole range. For these reasons, the hyperelastic material model was adopted as the physical model of the resin. The hyperelastic material model, used in the present paper, was described in Section 4. The physical constants for the analyzed resin are presented in Section 5.

In order to describe the response of the material filling the expansion joint in the beam, a three-dimensional *FEM* model is assumed. In contrast to the surface girder, where the dilatation gap is a long narrow structure, the plane strain problem is the most realistic physical model. Therefore, in the case of the plate, a two-dimensional model is considered.

The ABAQUS/CAE system was used for all the numerical simulations [20].

## 3. Description of Analyzed Material

The material analyzed in the numerical simulations was one-component resin based on polyurethane. This resin was selected because it is used for, among other things, injecting cracks and joints in reinforced concrete structures, repairing water leakages from expansion joints [9,10,11,12], the preventive sealing of structures, water infiltration control during tunnelling, curtain injections, injection repairs of concrete, and masonry underground structures (in basements, underground car parks, etc.).

The resin used in the tests is made up of two components: component A, polyurethane resin, and component B, water. The two components are mixed at the volumetric proportion of 1:1.

The components of the tested resin and its properties are presented in, respectively, Table 1 and Table 2.

## 4. Hyperelastic Material Model

The hyperelastic material is described by strain energy potential function U expressed per unit reference volume, enabling one to formulate a constitutive law [21,22]. For isotropic materials, the strain energy potential depends on the strain invariants only. It is possible to use different invariant sets in analytical description. The common choice is deviatoric strain invariants: ${\bar{I}}_{1},\;{\bar{I}}_{2}$, and elastic volume ratio $J_{el}$, which allow definition of U components responsible for deviatoric and volumetric strain parts:

(1) $$U=U_{dev}\left({\bar{I}}_{1},\;\;{\bar{I}}_{2}\right)+U_{vol}\left(J_{el}\right)$$

When thermal strains are absent, the elastic volume ratio $J_{el}$ is equal to total volume ratio J and can be expressed by the Jacobian of the transformation between the reference X and the current x configurations:

(2) $$F=\frac{\partial x}{\partial X},\;J_{el}=J=\det\left(F\right)=\frac{dV}{dV_{0}}$$

The deviatoric strain invariants can be expressed by deviatoric stretches ${\;\bar{\lambda }}_{i}$, principal stretches $\lambda_{i}$, or principal strains $\varepsilon_{i}$:

(3) $${\bar{I}}_{1}={\;\bar{\lambda }}_{1}^{2}+{\;\bar{\lambda }}_{2}^{2}+{\;\bar{\lambda }}_{3}^{2}$$

(4) $${\bar{I}}_{2}={\;\bar{\lambda }}_{1}^{-2}+{\;\bar{\lambda }}_{2}^{-2}+{\;\bar{\lambda }}_{3}^{-2}$$

(5) $${\;\bar{\lambda }}_{i}=J^{-\frac{1}{3}}\lambda_{i}=J^{-\frac{1}{3}}\left(1+\varepsilon_{i}\right)$$

ABAQUS offers several forms of strain energy potential U. In the present work, the Ogden model was applied to describe analysed material physical properties. Strain energy potential for considered form is defined by strain invariants and material coefficients [20,23,24]:

(6) $$U=\overset{N}{\underset{i=1}{\sum }}\frac{2\mu_{i}}{\alpha_{i}^{2}}\left({\bar{\lambda }}_{1}^{\alpha_{i}}+{\bar{\lambda }}_{2}^{\alpha_{i}}+{\bar{\lambda }}_{3}^{\alpha_{i}}-3\right)+\overset{N}{\underset{i=1}{\sum }}\frac{1}{D_{i}}{\left(J-1\right)}^{2i}$$

Material coefficients: $N_{i},\;\mu_{i},\;\alpha_{i}$, and $D_{i}$, which appear in the Ogden form, are related to engineering constants. The initial value of shear modulus $\mu_{i}$ and bulk modulus $K_{0}$ are expressed as follows:

(7) $$\mu_{0}=\overset{N}{\underset{i=1}{\sum }}\mu_{i},\;\;\;\;\;\;\;\;\;\;\;\;\;K_{0}=\frac{2}{D_{1}}.$$

## 5. Material Physical Constants

In paper [19], the authors described an algorithm for identifying the physical model for hyperelastic material and the decomposing of the associated physical constants. For the resin described in paragraph 3, the best compliance with the experimental results was obtained for the Ogden form for the strain energy potential order N=2. The physical constants derived for the considered material are collected in Table 3.

The parameters listed in the table above will be used to define material physical model in examples presented in next paragraphs.

## 6. Dilatation Gap Simulation in Beam: Three-Dimensional Problem

### 6.1. Physical and Discrete Models

**Figure 1:**
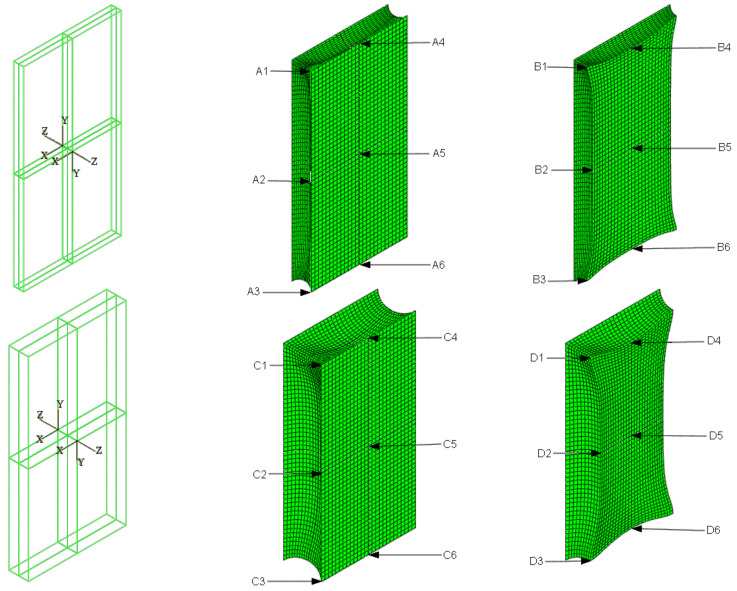
Material in dilatation gap numerical model. Three-dimensional problem. Expansion joint geometry and local coordinate systems on connection planes, left graphics. Selected nodes on connection plane, central graphics, and on central plane, right graphics. The width of the gap is equal to 1.0 cm, upper, and 2.0 cm, lower.

A rectangular beam measuring 10 × 20 cm with an expansion joint 1.0 or 2.0 cm wide is analyzed. In order to simulate an answer of the material in the dilatation gap, *FEM* discrete models were created (Figure 1). It is assumed that the concrete walls behave as perfectly rigid planes and the connection between the materials is ideal. Taking this it into account, stiff translations and rotations of the connection plane are applied as external kinematic loads. To be able to compare results for both widths, the loads ratio is the same and it is equal to 1:2.

### 6.2. Axial Stretching

**Figure 2:**
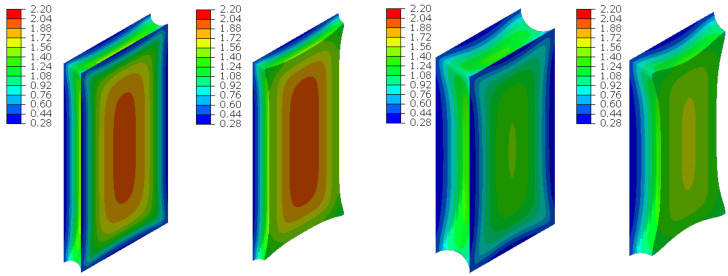
Deformations for scaling factor 1.0 and Cauchy stresses $\sigma_{z}$ in MPa for total load level. The width of the gap is equal to 1.0 cm, left graphics, and 2.0 cm, right graphics. Views on connection, first and third columns, or central plane, second and fourth columns.

The material in the dilatation gap is analyzed under uniform stretching equal to 1.0 or 2.0 cm in the *z*-axis direction for widths of 1.0 or 2.0 cm, respectively. Displacements $u_{z}=0.5$ or 1.0 cm, expressed in local coordinate systems of walls, are put on front and back connection planes (Figure 1). Deformation images for total load level are presented in Figure 2.

ABAQUS calculates Cauchy stresses (real stresses), expressed per unit deformed area. The nominal stresses, expressed per unit undeformed surface, differ significantly from the real stresses when the body in question is subjected to large deformations. In this case, the Cauchy stress values determine the stress intensity in the material. Figure 2 shows the Cauchy stresses $\sigma_{z}$ generated in the resin for total load level for both gap widths considered.

**Figure 3:**
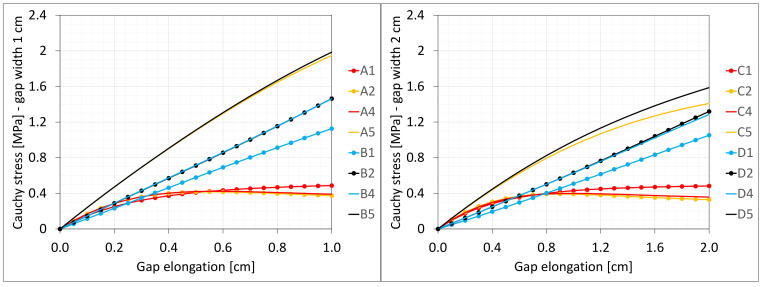
Equilibrium paths for stresses σ_z in selected nodes. The width of the gap is equal to 1.0 cm, left graphic, and 2.0 cm, right.

Figure 3 presents the same stresses $\sigma_{z}$ in selected nodes (Figure 1) versus the gap elongation for both widths considered.

**Figure 4:**
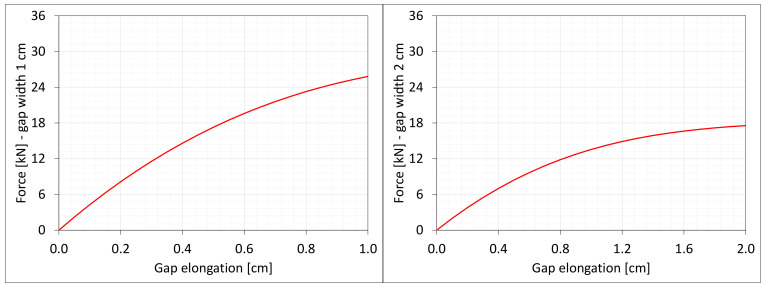
Equilibrium paths for excitation force $\sigma_{z}$ The width of the gap is equal to 1.0 cm, left graphic, and 2.0 cm, right graphic.

Figure 4 shows the total axial force $N_{z}$ generated in the resin versus the gap elongation. The normal stresses along the direction of excitation have different values in different points of the discrete model (Figure 3). Moreover, due to the differences in deformations (Figure 2) between the considered gap widths the stress values in the same nodes of the *FEM* model differ significantly from each other. Therefore, in order to compare the results, the excitation force values are included in the charts (Figure 4).

### 6.3. Axial Compression

**Figure 5:**
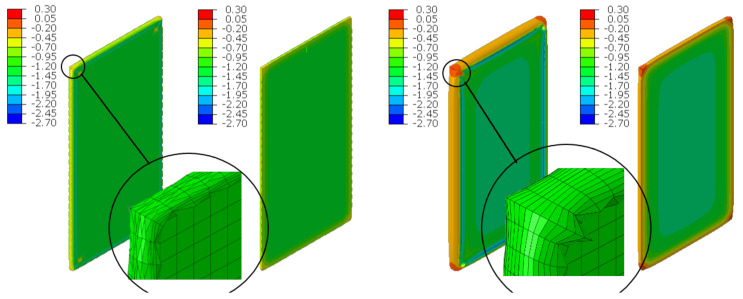
Deformations for scaling factor 1.0 and Cauchy stresses $\sigma_{z}$ in MPa for final load level. The width of the gap is equal to 1.0 cm, left graphic, and 2.0 cm, right graphic. Views on connection, first and third columns, or central plane, second and fourth columns. Details of deformation are enlarged.

The material in the dilatation gap is analyzed under uniform compression equal to 0.5 or 1.0 cm in the *z*-axis direction. Displacements $u_{z}=$ −0.25 or −0.5 cm, expressed in local coordinate systems of walls, are put on front and back connection planes (Figure 1). Deformation images and the Cauchy stresses $\sigma_{z}$ generated in the resin for final load level for both gap widths considered are presented in Figure 5.

The incremental algorithm used to solve the nonlinear problem modelled by *FEM* loses convergence when it is impossible to satisfy the equilibrium equations in the next iteration step. In the presented examples, it was impossible to reach total declared excitations; the convergence was achieved for shortenings not greater than 0.45 and 0.95 cm. Moreover, earlier than such final load levels are achieved, non-physical deformations appear (Figure 5) for shortenings greater than 0.375 and 0.65 cm.

**Figure 6:**
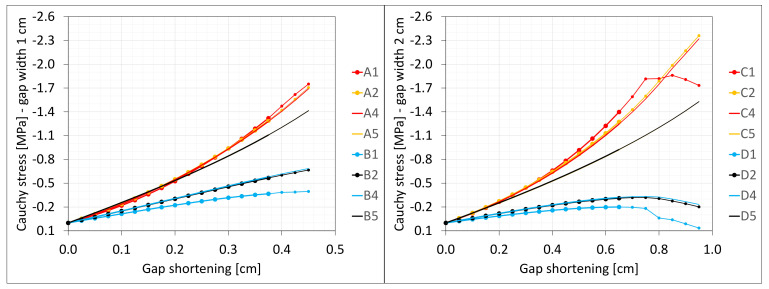
Equilibrium paths for stresses $\sigma_{z}$ in selected nodes. The width of the gap is equal to 1.0 cm, left graphic, and 2.0 cm, right graphic. Parts of charts with non-physical deformations are displayed with thin lines.

**Figure 7:**
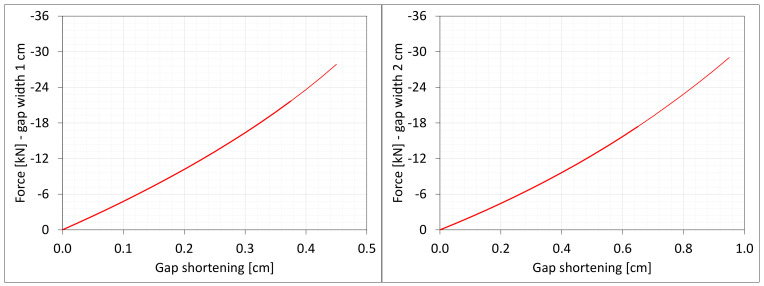
Equilibrium paths for excitation force $N_{z}$. The width of the gap is equal to 1.0 cm, left graphic, and 2.0 cm, right graphic. Parts of charts with non-physical deformations are displayed with thin lines.

The Cauchy stresses $\sigma_{z}$ in selected nodes (Figure 1) and the total axial force $N_{z}$ generated in the resin versus the gap shortening are shown in the Figure 6 and Figure 7, respectively. In the charts, the equilibrium paths parts, for which non-physical deformations appear, are displayed with thin lines.

### 6.4. Bending

**Figure 8:**
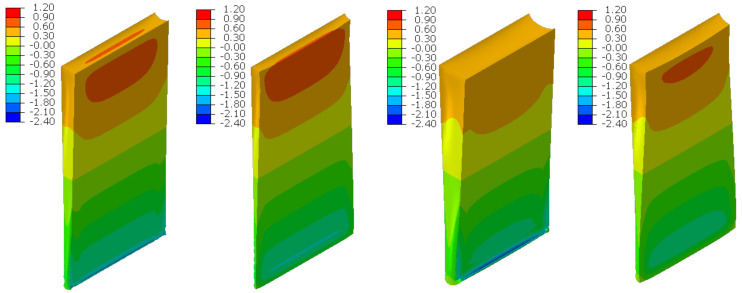
Deformations for scaling factor 1.0 and Cauchy stresses $\sigma_{z}$ in MPa for final load level. The width of the gap is equal to 1.0 cm, left graphics, and 2.0 cm, right graphics. Views on connection, first and third columns, or central plane, second and fourth columns.

**Figure 9:**
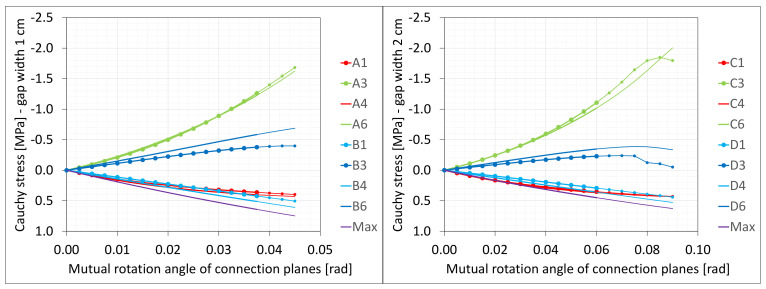
Equilibrium paths for stresses $\sigma_{z}$ in selected nodes. The gap width is equal to 1.0 cm, left graphic, and 2.0 cm, right graphic. Parts of charts with non-physical deformations are displayed with thin lines.

**Figure 10:**
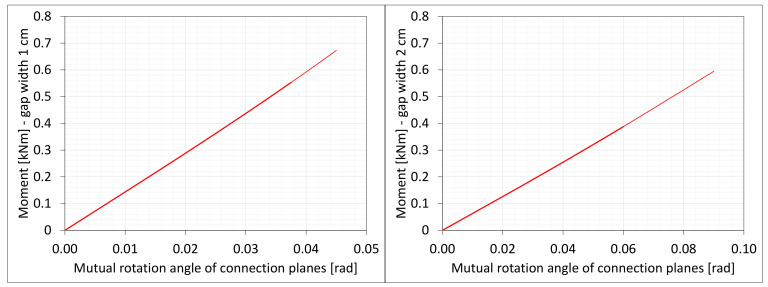
Equilibrium paths for excitation moment$\;M_{z}$. The gap width is equal to 1.0 cm, left graphic, and 2.0 cm, right graphic. Parts of charts with non-physical deformations are displayed with thin lines.

The material in the dilatation gap exposed to bending is analyzed. Rotations around the *x*-axis of local walls coordinate systems of $\phi_{x}$ = −0.025 or −0.05 rad and 0.025 or 0.05 rad are put on front and back connection planes, respectively (Figure 1). Assumed angles of rotation generate the same shortenings in beam bottom fibers, such as in the axial compression case considered. Deformation images and the Cauchy stresses $\sigma_{z}$ generated in the resin for final load level are presented in Figure 8. The same stresses $\sigma_{z}$ in selected nodes (Figure 1) and the total bending moment $M_{x}$ generated in the resin versus mutual rotation angle of the opposite connection planes are shown in Figure 9 and Figure 10, respectively. The stress distributions shown in Figure 8 indicate that the greatest tensile stresses will occur in different nodes than those shown in Figure 1. The equilibrium paths for these stresses are also shown in Figure 9. The convergence was achieved for mutual rotation angles not greater than 0.045 and 0.09 rad; non-physical deformations were detected for angles greater than 0.0375 and 0.06 rad.

**Figure 11:**
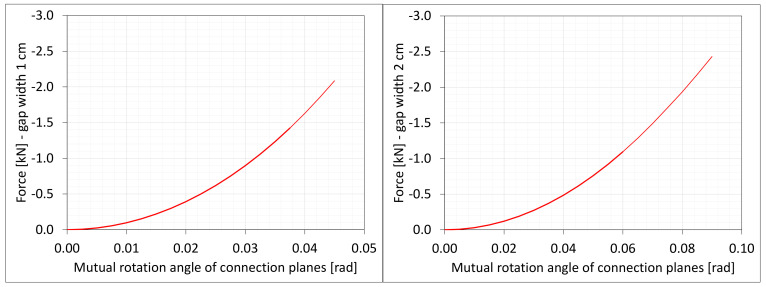
Equilibrium paths for excitation force $N_{z}$. The width of the gap is equal to 1.0 cm, left graphic, and 2.0 cm, right graphic. Parts of charts with non-physical deformations are displayed with thin lines.

Due to the different behavior of materials under stretching and compression (Figure 8) in the resin, which fills expansion joint, axial force is generated in addition. The equilibrium path for the force $N_{z}$ is displayed in Figure 11.

### 6.5. Shearing

**Figure 12:**
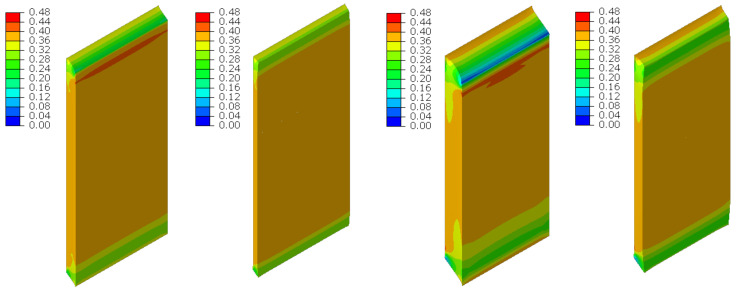
Deformations for scaling factor 1.0 and Cauchy stresses $\tau_{zy}$ in MPa for total load level. The width of the gap is equal to 1.0 cm, left graphics, and 2.0 cm, right graphic. Views on connection, first and third columns, or central plane, second and fourth columns.

**Figure 13:**
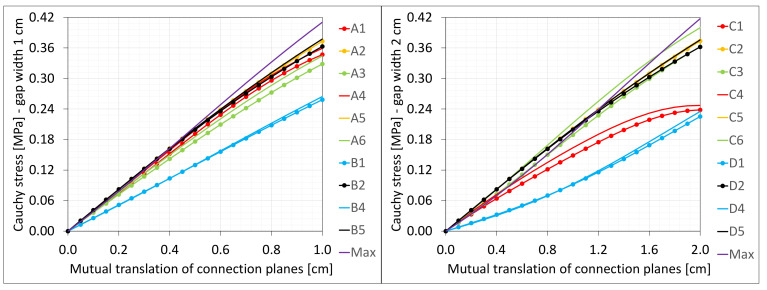
Equilibrium paths for stresses $\tau_{zy}$ in selected nodes. The width of the gap is equal to 1.0 cm, left graphic, and 2.0 cm, right graphic.

**Figure 14:**
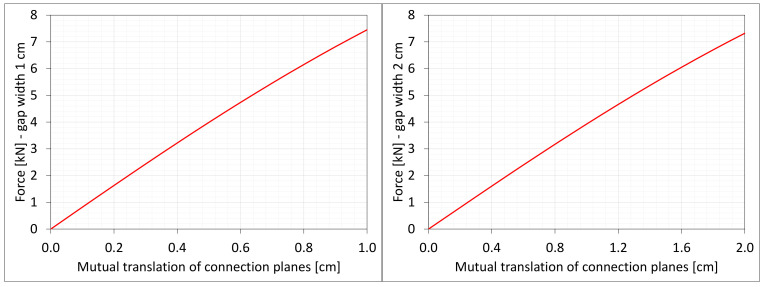
Equilibrium paths for shear force $V_{y}$. The width of the gap is equal to 1.0 cm, left graphic, and 2.0 cm, right graphic.

**Figure 15:**
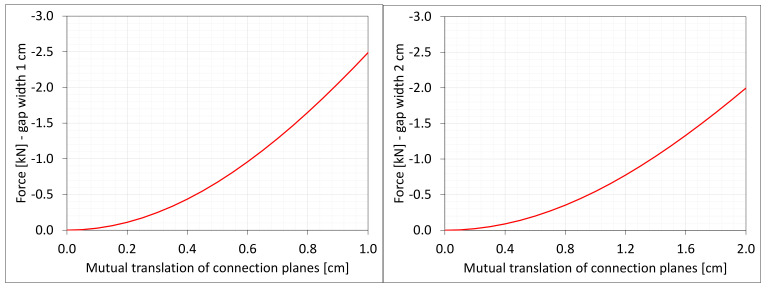
Equilibrium paths for axial force $N_{z}$. The width of the gap is equal to 1.0 cm, left graphic, and 2.0 cm, right graphic.

The material in the dilatation gap exposed to shearing is analyzed. Displacements $u_{y}$ = 0.5 or 1.0 cm, expressed in local coordinate systems of walls, are put on front and back connection planes (Figure 1). Deformation images and the Cauchy stresses $\tau_{zy}$ generated in the resin for total load level are presented in Figure 12. The same stresses $\tau_{zy}$ in selected nodes (Figure 1) and the total shear force $V_{y}$ generated in the resin versus mutual translation of the opposite connection planes are shown in Figure 13 and Figure 14, respectively. The equilibrium paths for the greatest shear stresses, which occurred in different nodes than those shown in Figure 1, are also displayed in Figure 13. Additionally, the axial force $N_{z}$ is generated in the resin. The equilibrium paths for these forces are displayed in Figure 15.

## 7. Dilatation Gap Simulation in Plate: Plane Strain Problem

### 7.1. Physical and Discrete Models

**Figure 16:**
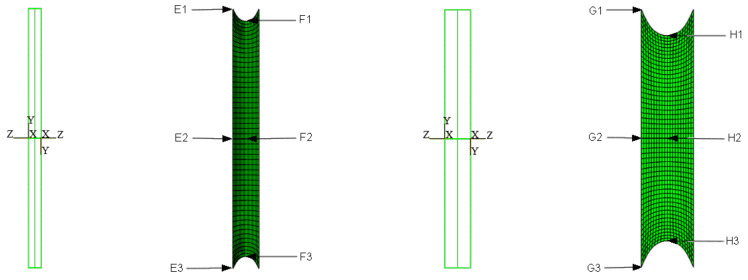
Material in dilatation gap numerical model. Plane strain problem. Expansion joint geometry and local coordinate systems on connection planes, first and third graphics. Selected nodes on connection and central plane, second and fourth graphics. The width of the gap is equal to 1.0 cm, left, and 2.0 cm, right.

In order to compare the current results with the values obtained in paragraph 6, a plate thickness equal to the height of the analyzed beam was assumed to be 20 cm, and the same expansion joint widths equal to 1.0 or 2.0 cm were considered (Figure 16). The kinematic assumptions and load ratio for both gap widths applied for the three-dimensional discrete model are the same for the considered plane problem.

### 7.2. Axial Stretching

**Figure 17:**
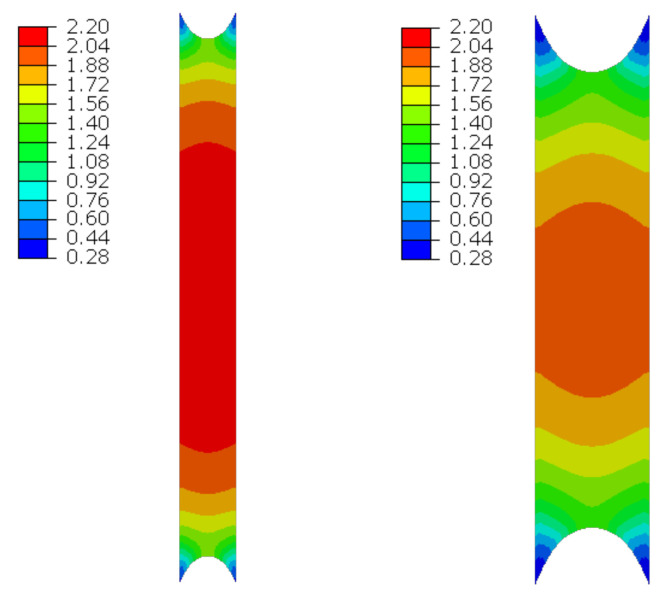
Deformations for scaling factor 1.0 and Cauchy stresses $\sigma_{z}$ in MPa for total load level. The width of the gap is equal to 1.0 cm, left graphic, and 2.0 cm, right graphic.

The material in the dilatation gap is analyzed under uniform stretching equal to 1.0 or 2.0 cm in the *z*-axis direction. Displacements $u_{z}$= 0.5 or 1.0 cm, expressed in local coordinate systems of walls, are put on left and right connection planes (Figure 16). Deformation images and the Cauchy stresses $\sigma_{z}$ in the resin for total load level are presented in Figure 17.

**Figure 18:**
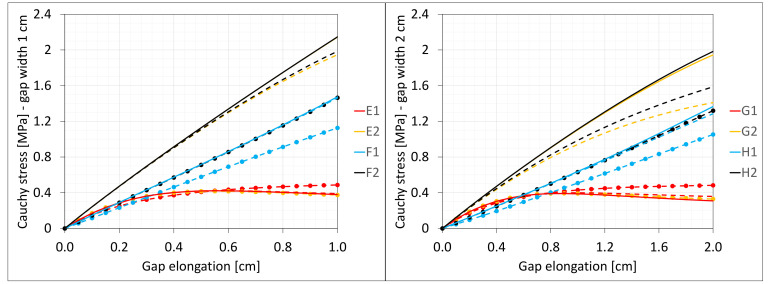
Equilibrium paths for stresses $\sigma_{z}$ in selected nodes. The width of the gap is equal to 1.0 cm, left graphic, and 2.0 cm, right graphic. Plane strain models, solid lines; and 3D models, dashed lines (curves from Figure 3).

**Figure 19:**
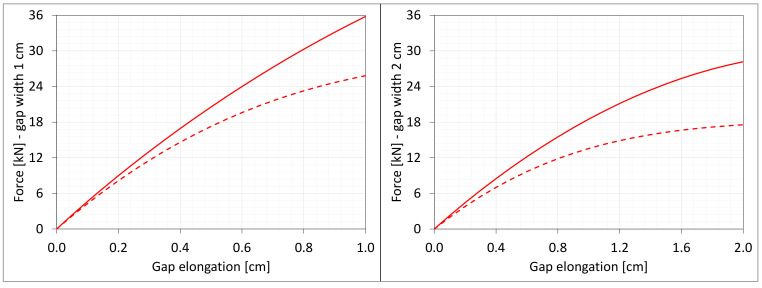
Equilibrium paths for excitation force $N_{z}$. The width of the gap is equal to 1.0 cm, left graphic, and 2.0 cm, right graphic. Plane strain models, solid lines; and 3D models, dashed lines (curves from Figure 4).

Figure 18 and Figure 19 present the same stresses $\sigma_{z}$ in selected nodes (Figure 16) and the excitation force $N_{z}$ versus the gap elongation, respectively. The force is from the same area as for the paths shown in Figure 4. For comparison, the results from the discrete 3D model are shown in the background. Curves for nodes lying on the same planes and with the same coordinates *z* are displayed in the same color (Figure 18).

### 7.3. Axial Compression

**Figure 20:**
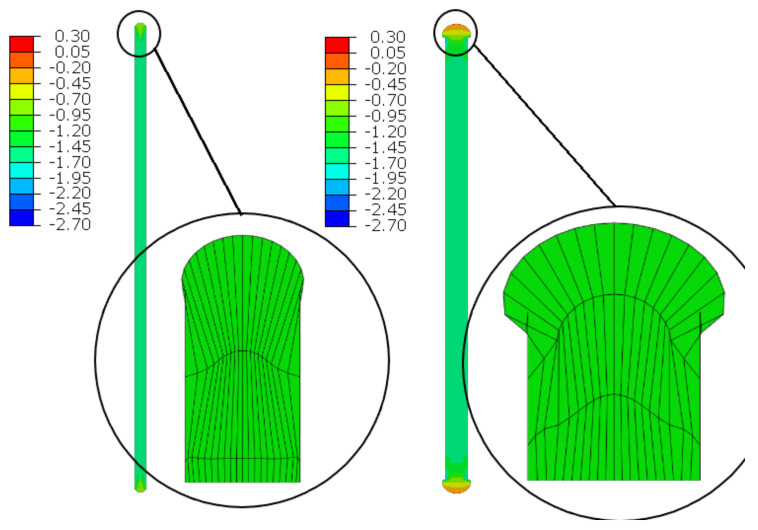
Deformations for scaling factor 1.0 and Cauchy stresses $\sigma_{z}$ in MPa for total load level. The width of the gap is equal to 1.0 cm, left graphic, and 2.0 cm, right graphic. Details of deformation are enlarged.

**Figure 21:**
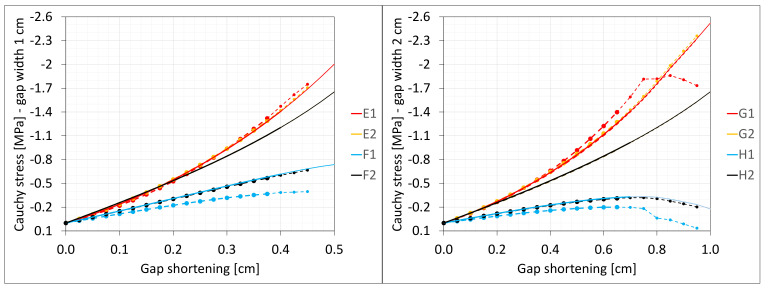
Equilibrium paths for stresses $\sigma_{z}$ in selected nodes. The width of the gap is equal to 1.0 cm, left graphic, and 2.0 cm, right graphic. Plane strain models, solid lines; and 3D models, dashed lines (curves from Figure 6). Parts of charts with non-physical deformations are displayed with thin lines.

**Figure 22:**
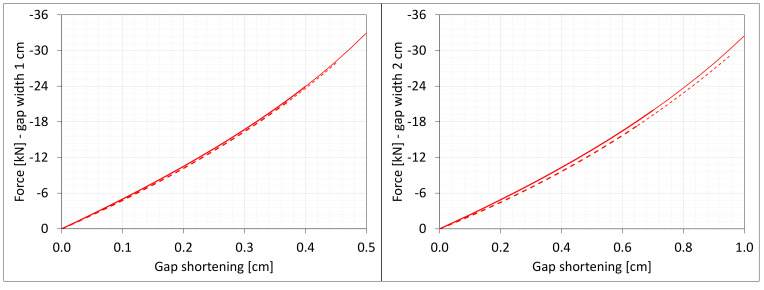
Equilibrium paths for excitation force $N_{z}$ The width of the gap is equal to 1.0 cm, left graphic, and 2.0 cm, right. Plane strain models, solid lines; and 3D models, dashed lines (curves from Figure 7). Parts of charts with non-physical de-formations are displayed with thin lines.

One analyses the material in the dilatation gap under uniform compression equal to 0.5 or 1.0 cm in the *z*-axis direction. Displacements $u_{z}$ = −0.25 or −0.5 cm, expressed in local coordinate systems of walls, are put on left and right connection planes (Figure 16). Deformation images and the Cauchy stresses $\sigma_{z}$ generated in the resin for total load level are presented in Figure 20. The same stresses $\sigma_{z}$ in selected nodes (Figure 16) and the total axial force $N_{z}$ generated in the resin versus the gap shortening are shown in Figure 21 and Figure 22, respectively. The convergence was achieved in the whole excitation ranges, and non-physical deformations (Figure 20) were detected for shortenings greater than 0.4 and 0.7 cm.

### 7.4. Bending

**Figure 23:**
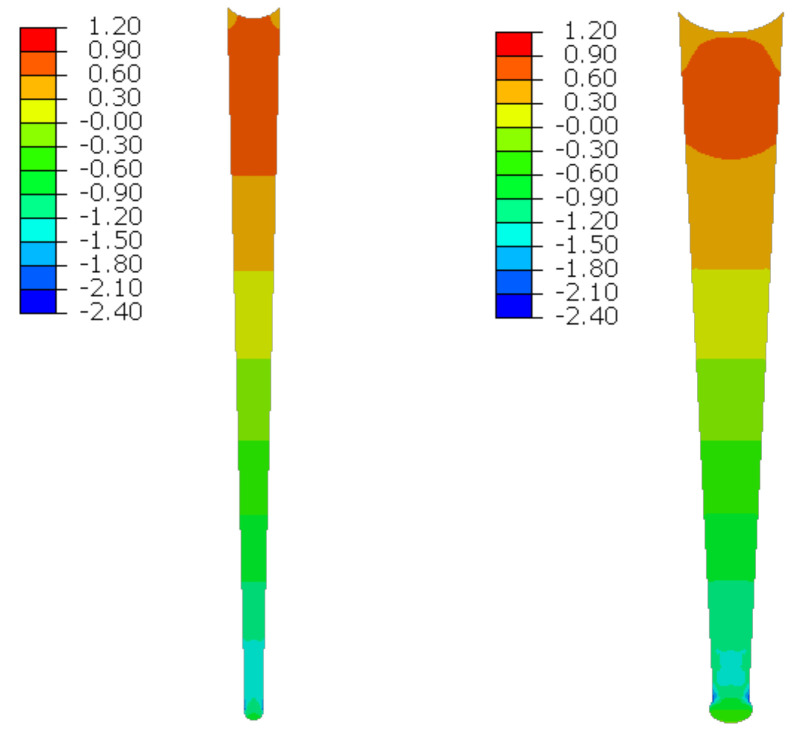
Deformations for scaling factor 1.0 and Cauchy stresses $\sigma_{z}$ in MPa for total load level. The width of the gap is equal to 1.0 cm, left graphic, and 2.0 cm, right graphic.

**Figure 24:**
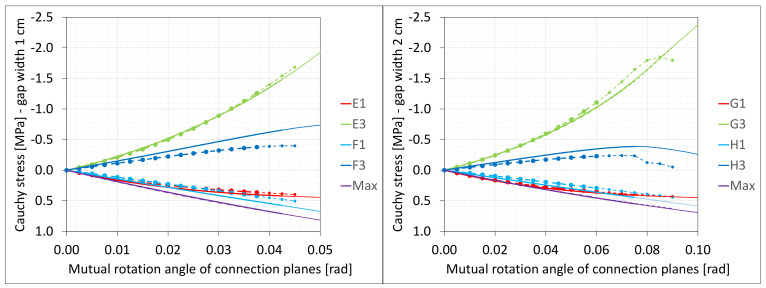
Equilibrium paths for stresses $\sigma_{z}$ in selected nodes. The width of the gap is equal to 1.0 cm, left graphic, and 2.0 cm, right graphic. Plane strain models, solid lines; and 3D models, dashed lines (curves from Figure 9). Parts of charts with non-physical deformations are displayed with thin lines.

**Figure 25:**
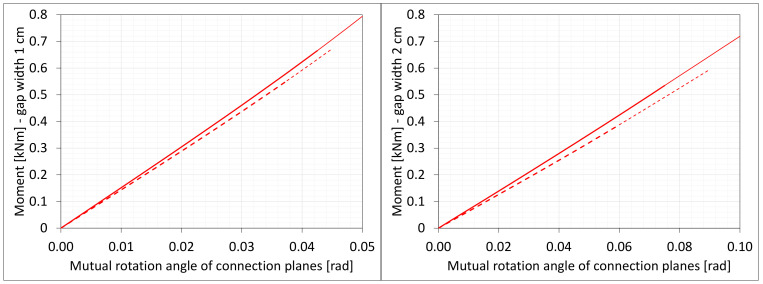
Equilibrium paths for excitation moment $M_{z}$. The width of the gap is equal to 1.0 cm, left graphic, and 2.0 cm, right graphic. Plane strain models, solid lines; and 3D models, dashed lines (curves from Figure 10). Parts of charts with non-physical deformations are displayed with thin lines.

**Figure 26:**
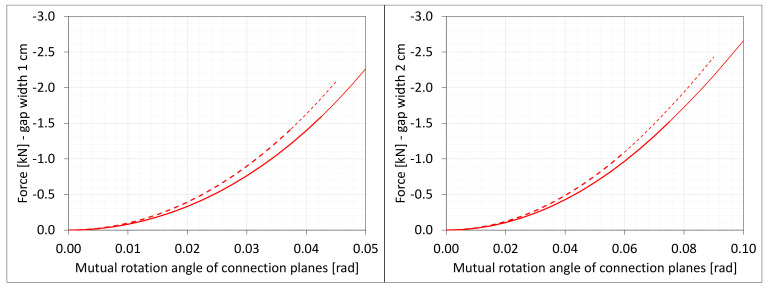
Equilibrium paths for axial force $N_{z}$. The width of the gap is equal to 1.0 cm, left graphic, and 2.0 cm, right graphic. Plane strain models, solid lines; and 3D models, dashed lines (curves from Figure 11). Parts of charts with non-physical deformations are displayed with thin lines.

The material in the dilatation gap exposed to bending are analyzed. Rotations around the *x*-axis of local walls coordinate systems of $\phi_{x}$ = −0.025 or −0.05 rad and 0.025 or 0.05 rad are put on left and right connection planes, respectively (Figure 16). Deformation images and the Cauchy stresses $\sigma_{z}$ generated in the resin for total load level are presented in Figure 23. The same stresses $\sigma_{z}$ in selected nodes (Figure 16) and the total bending moment $M_{x}$ generated in the resin versus mutual rotation angle of the opposite connection planes are shown in Figure 24 and Figure 25, respectively. The equilibrium paths for the greatest tensile stresses, which occurred in different nodes than those shown in Figure 16, are also displayed in Figure 24. The convergence was achieved in the whole excitation range; non-physical deformations were detected for angles greater than 0.0425 and 0.075 rad. The equilibrium path for the force $N_{z}$ is displayed in Figure 26.

### 7.5. Shearing

**Figure 27:**
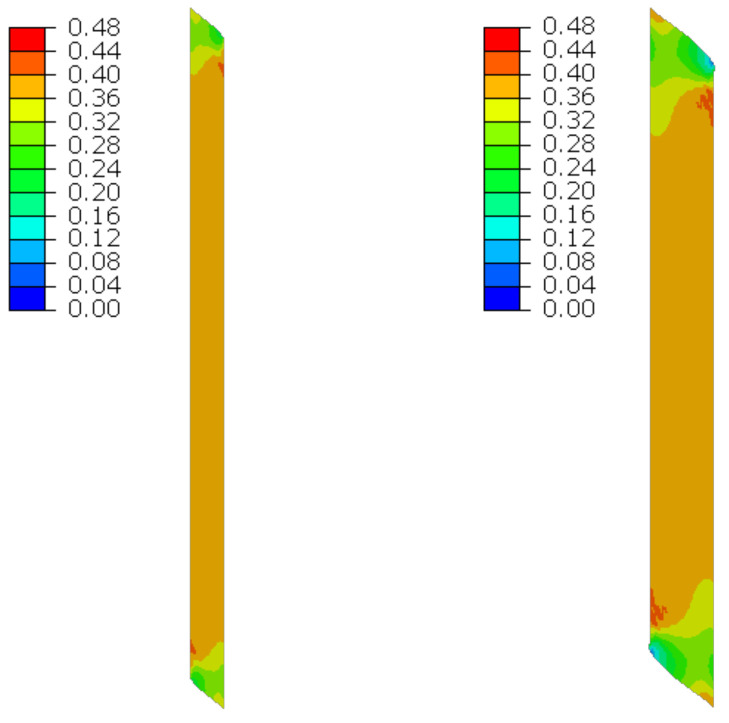
Deformations for scaling factor 1.0 and Cauchy stresses $\tau_{zy}$ in MPa for total load level. The width of the gap is equal to 1.0 cm, left graphic, and 2.0 cm, right graphic.

**Figure 28:**
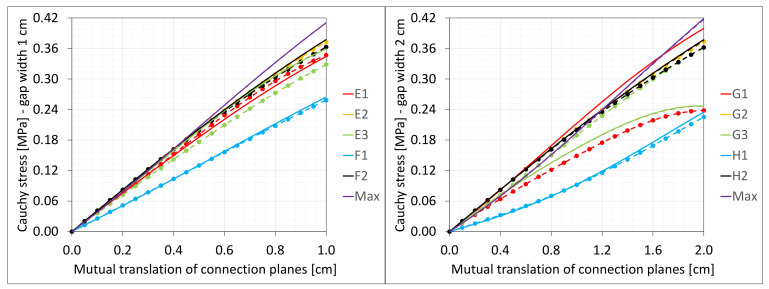
Equilibrium paths for stresses $\tau_{zy}$ in selected nodes. The width of the gap is equal to 1.0 cm, left graphic, and 2.0 cm, right graphic. Plane strain models, solid lines; and 3D models, dashed lines (curves from Figure 13).

**Figure 29:**
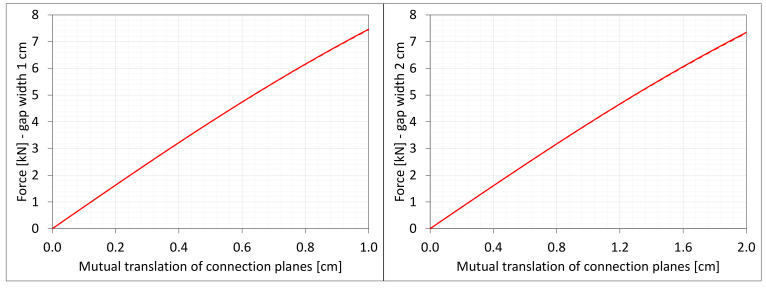
Equilibrium paths for shear force $V_{y}$. The width of the gap is equal to 1.0 cm, left graphic, and 2.0 cm, right graphic. Plane strain models, solid lines; and 3D models, dashed lines (curves from Figure 14).

**Figure 30:**
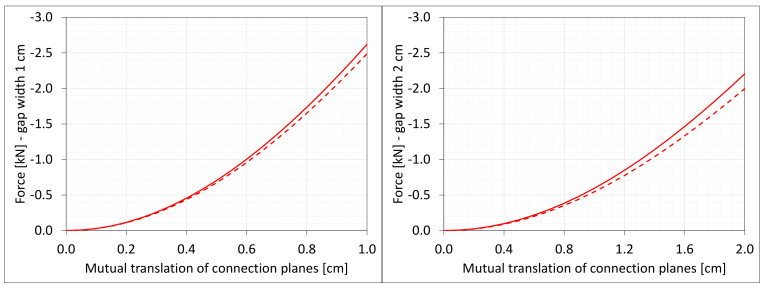
Equilibrium paths for axial force $N_{z}$. The width of the gap is equal to 1.0 cm, left graphic, and 2.0 cm, right graphic. Plane strain models, solid lines; and 3D models, dashed lines (curves from Figure 15).

The material in the dilatation gap exposed to shearing is analyzed. Displacements $u_{y}$ = 0.5 or 1.0 cm, expressed in local coordinate systems of walls, are put on left and right connection planes (Figure 16). Deformation images and the Cauchy stresses $\tau_{zy}$ generated in the resin for total load level are presented in Figure 27. The same stresses $\tau_{zy}$ in selected nodes (Figure 16) and the total shear force $V_{y}$ generated in the resin versus mutual translation of the opposite connection planes are shown in Figure 28 and Figure 29, respectively. The equilibrium paths for the greatest shear stresses, which occurred in different nodes than those shown in Figure 16, are also displayed in Figure 28. The equilibrium paths for the axial force $N_{z}$, which are generated in the resin, are displayed in Figure 30.

## 8. Results Discussion

The results of the numerical analyses presented in the previous chapters allow for the formulation of several general observations. In the considered ranges of deformations, clearly nonlinear forms of equilibrium paths are noted. In the case of axial stretching, the stresses and internal forces caused by the leading deformation of the same value are greater in a gap of a smaller width, for axial compression they are smaller, and in other cases, no clear tendency is observed. The differences in the global response between the three- and two-dimensional models are insignificant, except in the case of axial stretching (force $N_{z}$ in Figure 19).

For two-dimensional models, the *FEM* algorithm achieves convergence in a larger range of forcing, later non-physical deformations appear. The effect of non-physical deformations on the global response is not noticed, the effects of their occurrence are visible only on stress equilibrium paths in nearby nodes.

The results obtained in the numerical simulations are in expected and acceptable ranges. This suggests that physical model of the consider material, which was identified in the previous stage of investigations, is proper. A more categorical statement, however, would require empirical confirmation, which is the future intention of the authors.

## 9. Conclusions

Due to the physical nonlinearity and high strains, adopting a hyperelastic material model for the analyzed resin is the right choice. The experience gained during the analysis of the presented examples indicates that the results are highly sensitive to changes in the values of physical constants (Table 3). Therefore, they should be determined with high accuracy.

Using *FEM* simulations one can determine the values of stresses and cross-sectional forces in the filling material, which can be the basis for the design of expansion joints geometry. Each of the analyzed elementary deformations generates a complex stress state in the material filling the expansion joint. In the present study, the authors suggest that it is possible to determine reasonable numerical stress values for different excitations in the case of the investigated material. 

As part of further research, the authors’ aim will be to formulate criteria for the damage of the material and breaking its connection with the reinforced concrete structure and their experimental verification. The results of these analyses could form the basis of guidelines for the design of expansion joint gaps filled with material, ensuring the waterproofing of the joint.

## Figures and Tables

**Table 1:** Components of tested resin.

Parameter	Component A	Component B
Description	Polyurethane base	Water
Form	Liquid	Liquid
pH	Undetermined	7
Density	from 1.04 to 1.16 kg/dm^3^	ca. 1.00 kg/dm^3^
Viscosity	<350 mPas	ca. 1.00 mPas

**Table 2:** Properties of tested resin.

Property	Value
Viscosity	<200 mPas
Foam factor	>3
Tensile strength	approx. 1.3 MPa
Elongation at break	approx. 160%

**Table 3:** Material coefficients.

N	$\mu_{1}$	$\mu_{2}$	$\alpha_{1}$	$\alpha_{2}$	$D_{1}$	$D_{2}$	$\mu_{0}$	$K_{0}$
[-]	[kPa]	[kPa]	[-]	[-]	[MPa^−1^]	[MPa^−1^]	[kPa]	[kPa]
2	410.2	3.306	1.218	−2.883	1.036	0.000	413.5	1930

## Data Availability

Not applicable.
